# Radical Surgery of Only the Anterior Elements of the Spine at the Posterior Element Fusion Level due to Metastatic Thyroid Cancer

**DOI:** 10.1155/2017/2365808

**Published:** 2017-06-27

**Authors:** Ryuto Tsuchiya, Kazuki Fujimoto, Kazuhide Inage, Sumihisa Orita, Yasuhiro Shiga, Hiroto Kamoda, Kazuyo Yamauchi, Miyako Suzuki, Jun Sato, Koki Abe, Hirohito Kanamoto, Masahiro Inoue, Hideyuki Kinoshita, Masaki Norimoto, Tomotaka Umimura, Masao Koda, Takeo Furuya, Junichi Nakamura, Kazuhisa Takahashi, Seiji Ohtori

**Affiliations:** ^1^Department of Orthopaedic Surgery, Graduate School of Medicine, Chiba University, Chiba, Japan; ^2^Department of Orthopaedic Surgery, Chiba Cancer Center, Chiba, Japan; ^3^Department of Orthopaedic Surgery, Chiba Aoba Municipal Hospital, Chiba, Japan

## Abstract

Spinal metastasis of differentiated thyroid cancer can have a favorable prognosis if radical surgery is performed. We encountered a case of spinal metastasis involving three anterior vertebral bodies at the posterior element fusion level and successfully achieved adequate stability by radical surgery involving only the anterior elements. A 67-year-old woman who had numbness and muscle weakness in the lower limbs caused by metastatic spinal tumor at the posterior element fusion level of L1–L3 vertebrae was treated with radical surgery of only the anterior element to gain stability. Similar situations may occur in cases involving other malignant tumor metastases or spinal primary tumors. If such a case occurs, this method could be useful in preventing metastasis to the posterior element.

## 1. Introduction

Differentiated thyroid carcinoma rarely metastasizes to the bone, especially to the spine, but a favorable surgical outcome can be obtained by radical surgery if metastasis is only present locally [1]. However, there has been no report of spinal metastasis occurring at the level of the posterior element fusion. Therefore, if the posterior element is involved, the appropriate surgical approach is still unknown.

We encountered a case of metastatic spinal tumor of thyroid cancer involving three anterior vertebral bodies at the posterior element fusion level and successfully achieved adequate stability by radical surgery of only the anterior element. This procedure included posterior fixation, resection of anterior vertebral bodies, and anterior reconstruction.

## 2. Case Presentation

A 67-year-old woman had previously undergone posterior fixation surgery (T12, L1, and L3) for an L2 compression fracture when she was 63 years old and had no other specific medical history. She had no symptoms after the surgery until the age of 67 years but developed numbness and muscle weakness of both her lower limbs 4 months ago and visited another doctor before visiting us.

Her previous doctor suspected metastatic spinal tumor based on the initial radiography and magnetic resonance imaging (MRI). She was diagnosed with metastases of thyroid follicular carcinoma by a subsequent bone biopsy and was referred to our hospital after total thyroidectomy 2 months before she visited us for the treatment of the spinal lesion.

**Figure 1:**
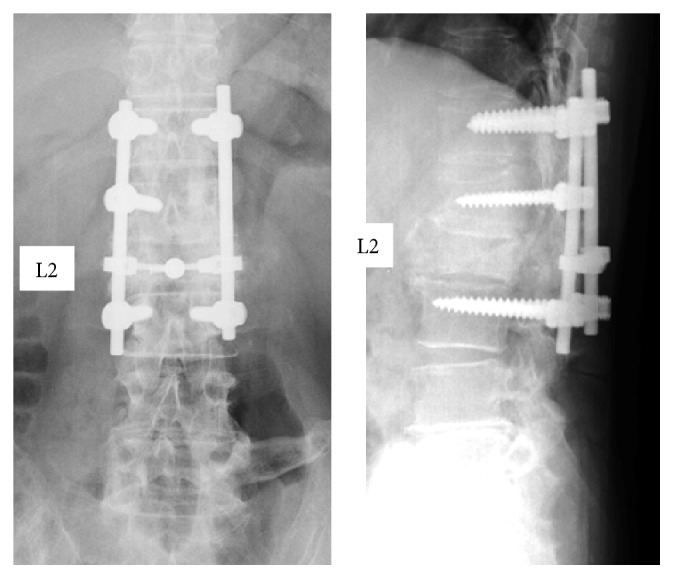
X-ray image of the spine at the first visit. Deformation of L2 vertebral body after posterior fixation surgery.

**Figure 2:**
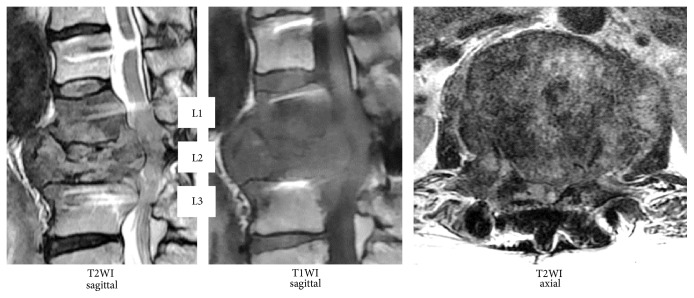
MRI findings. Brightness changes in the L1, L2, and L3 vertebral bodies and compression of the spinal canal by the tumor at the same level.

**Figure 3:**
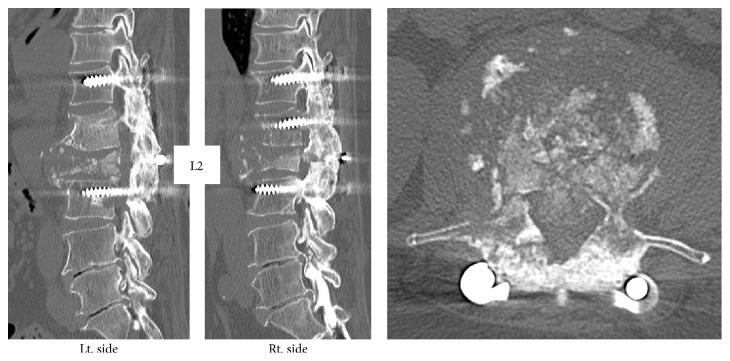
CT images. Vertebral arch (T12–L3) after posterior fixation surgery indicated complete bone union and bridge formation.

**Figure 4:**
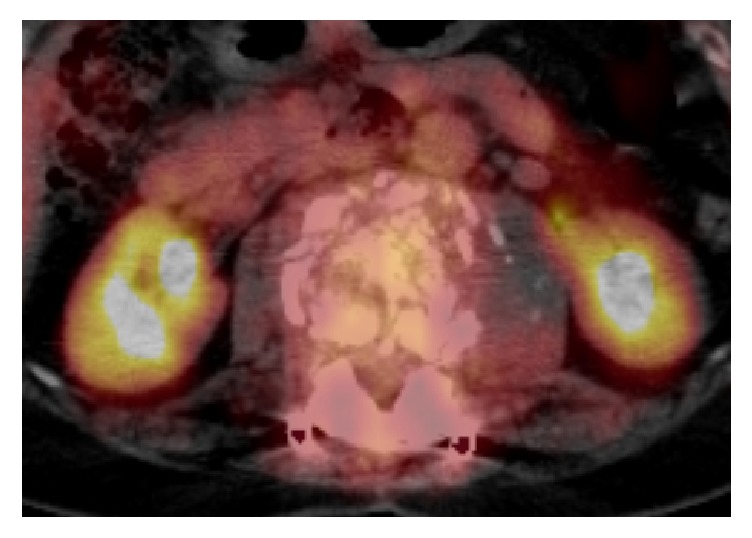
Uptake for metastases in the posterior element was not observed in PET/CT.

The patient's main complaints were numbness and muscle weakness of both lower limbs. We confirmed deformation of the L2 vertebral body by the initial radiography and MRI from the previous facility revealed hyperintensity/hypointensity of the L1, L2, and L3 vertebral bodies and compression of the spinal canal by the tumor at the same level (Figures 1 and 2). After posterior fixation surgery, the vertebral arch (T12–L3) in a CT scan indicated a complete bone union and bridge formation (Figure 3). PET/CT revealed no uptake in the posterior element, thus demonstrating no residual metastasis (Figure 4). Assessment of bone density by dual energy X-ray absorptiometry (DXA) indicated osteopenia in the femoral neck (*t* score: −1.7). Since the primary disorder was thyroid cancer without metastases in other viscera and bones except spinal vertebral bodies and the general condition of the patient was favorable (Performance Status grade 1), we decided to perform radical surgery according to the spinal metastatic tumor scoring system (Tokuhashi score 12 points/Tomita score 3 points) [2, 3]. We performed radical surgery by posterior fixation and excision of the anterior segment of the vertebral body and reconstructed the anterior segment via the posterior-anterior approach. Successful stability was achieved by maintaining the posterior element free of tumor. The surgery was performed as follows.

Prior to surgery, a radiologist performed transcatheter arterial embolization (TAE) of the trophic artery of the affected vertebral body.

At first, we performed the surgery with the patient in a prone position. After removing the posterior implant, we inserted pedicle screws into T11, T12, L4, and L5, fixed the rod, and performed posterior fusion surgery.

**Figure 5:**
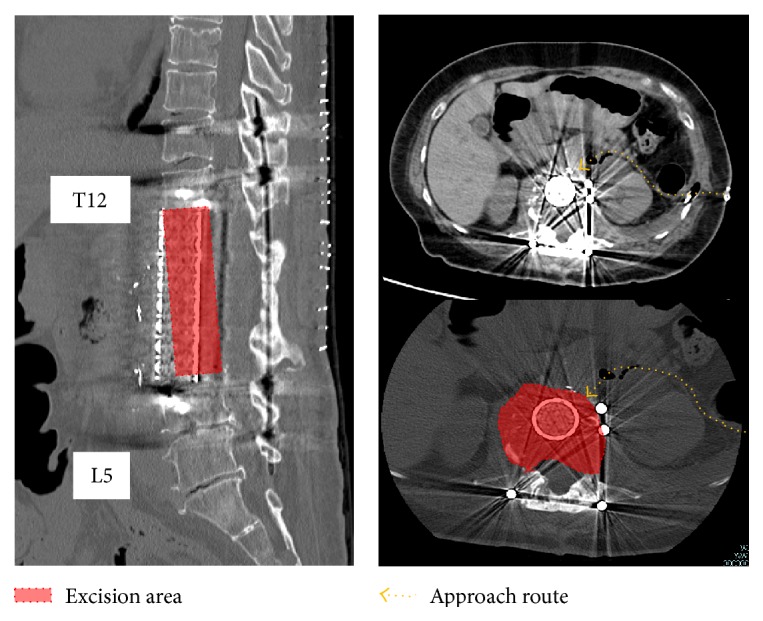
Postoperative CT images. The dashed line shows the approach route. The painted area indicates the excision area.

Subsequently, we placed the patient in right lateral decubitus position. We approached the anterior side of the vertebral body via extrapleural and extraperitoneal approach after resecting the 10th rib. We confirmed that there was no adhesion of abdominal large blood vessels and the tumor, liberated anterior aorta and inferior vena cava from the surrounding tissues, chiseled left and right pedicles, and removed L1–L3 anterior vertebral bodies, which were clumped with the tumor. Then, we set an appropriate length of both the mesh cage and autologous bone in the space after anterior element excision, inserted the screws into the T12 and L4 vertebral bodies, fastened the plate, and performed anterior fixation (Figure 5).

**Figure 6:**
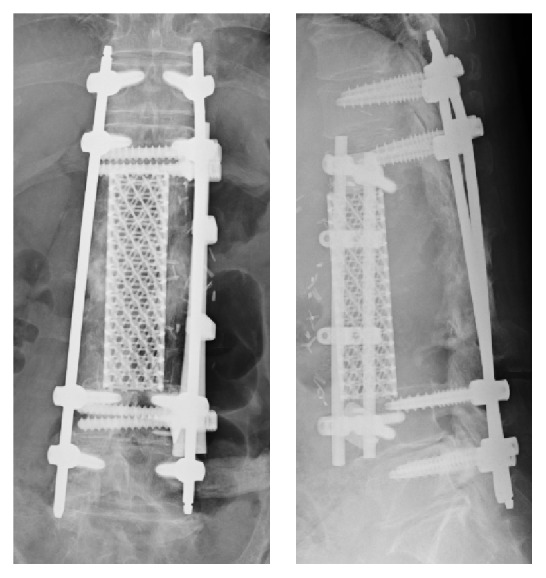
X-ray image of the spine after surgery.

The possibility of implant dislodgement due to osteopenia was addressed by performing short posterior fixation (2 above 2 below) and resection of the anterior affected portions of L1–L3 with the use of mesh cage and anterior plate (Figure 6). The patient has been doing well for 18 months after the surgery, without implant dislodgement or local recurrence.

## 3. Discussion

In the current case, neurological symptoms occurred as the primary symptoms when metastatic spinal tumor of thyroid cancer was found at the posterior element fusion level after posterior fixation. Then we experienced a favorable course resulting in adequate stability with short posterior fixation and resection/reconstruction of anterior element after confirming no metastasis to posterior element in such case. However, we highlight the following two clinical issues in the present case.

First, we encountered metastatic spinal tumors of thyroid cancer at the posterior element fusion level after spinal fusion with neurological symptoms occurring as the primary symptoms. According to previous reports, bone metastasis is found in 2–13% of differentiated thyroid cancer cases and spinal metastasis accounted for approximately 70% of them [4]. However, since only approximately 4% of distant metastases present with primary symptoms [5], it takes a long time to arrive at the right diagnosis and usually involves redundant medical examinations [6]. There has been a case report of spinal compression fracture presenting as the primary symptom without thyroid symptoms [6], but there is no report of spinal metastasis to the anterior element at the posterior element fusion level. In the present case, we could not get the images from the first surgery; therefore, we are assuming that there is a possibility that the initial vertebral compression fracture could be due to spinal metastasis of thyroid cancer and neurological symptoms might have appeared after synostosis of the posterior element due to a gradual progress.

Secondly, after confirming no metastasis to the posterior element, we could achieve adequate stability by the maintenance of the posterior element, short posterior fixation, and resection of the anterior element. Total en bloc spondylectomy (TES) can be an appropriate procedure in case of radical resection for spinal metastasis of thyroid cancer [7], but there is no report describing the use of radical surgery alone for anterior resection. Generally, spinal instability can occur in TES if spinal continuity is interrupted by removal of 3 spinal vertebral bodies [8, 9]. In such a case, reconstruction of the posterior element might be considered and reports indicate that such reconstruction was accomplished with the use of titanium mesh at the level of the thoracolumbar spine in adults [10]. One of the causes of instability in TES is the loss of the posterior element; therefore, we successfully achieved spinal stability by using short posterior fixation and resection of the anterior element because no metastases were identified in the posterior element.

In conclusion, spinal metastasis would possibly occur at posterior element fusion level in thyroid cancer, but a favorable prognosis along with spinal stability through resection/reconstruction of anterior element can be achieved if there are no metastases to the posterior element. This approach can be used in cases of other malignant metastases or spinal primary tumors, but only if metastasis to the posterior element is negative. However, further long-term studies are required with a large sample population and a long-term follow-up to corroborate the efficacy of this approach. Additionally, it is also necessary to collect more cases of malignant tumors at the posterior element fusion level and characterize the disorders that can be effectively treated by this surgery.

## References

[1] Bernier M.-O., Leenhardt L., Hoang C. (2001). Survival and therapeutic modalities in patients with bone metastases of differentiated thyroid carcinomas. *Journal of Clinical Endocrinology and Metabolism*.

[2] Tokuhashi Y., Matsuzaki H., Oda H., Oshima M., Ryu J. (2005). A revised scoring system for preoperative evaluation of metastatic spine tumor prognosis. *Spine*.

[3] Tomita K., Kawahara N., Kobayashi T., Yoshida A., Murakami H., Akamaru T. (2001). Surgical strategy for spinal metastases. *Spine*.

[4] Muresan M. M., Olivier P., Leclère J. (2008). Bone metastases from differentiated thyroid carcinoma. *Endocrine-Related Cancer*.

[5] Klubo-Gwiezdzinska J., Morowitz D., Van Nostrand D. (2010). Metastases of well-differentiated thyroid cancer to the gastrointestinal system. *Thyroid*.

[6] Kim D. H., Yoo S. D., Kim S. M., Im S. J., Kang J. K., Cho E. H. (2012). Thyroid cancer initially presenting compression fracture without common thyroid symptoms. *Annals of Rehabilitation Medicine*.

[7] Matsumoto M., Tsuji T., Iwanami A. (2013). Total en bloc spondylectomy for spinal metastasis of differentiated thyroid cancers: A long-term follow-up. *Journal of Spinal Disorders and Techniques*.

[8] Boriani S., de Lure F., Bandiera S. (2000). Chondrosarcoma of the mobile spine: report on 22 cases. *Spine*.

[9] Boriani S., Bandiera S., Donthineni R. (2010). Morbidity of en bloc resections in the spine. *European Spine Journal*.

[10] Chung J.-Y., Kim S.-K., Jung S.-T., Lee K.-B. (2013). New posterior column reconstruction using titanium lamina mesh after total en bloc spondylectomy of spinal tumour. *International Orthopaedics*.

